# Uterine Artery Embolization in a Patient With Large Uterine Fibroids

**DOI:** 10.7759/cureus.39740

**Published:** 2023-05-30

**Authors:** Safura Sattar, David Naimzadeh, Bita C Behaeddin, Ilya Fonarov, Damian Casadesus

**Affiliations:** 1 Research, California Institute of Behavioral Neurosciences & Psychology, Fairfield, USA; 2 Internal Medicine, Henry Ford Health System, Clinton Township, USA; 3 Medicine, St. George's University School of Medicine, Great River, USA; 4 Internal Medicine, Jackson Memorial Hospital, Miami, USA

**Keywords:** obstetrics and gynecology, interventional radiology, interventional radiology guided embolization, uterine fibroids, uterine leiomyoma, fibroids, leiomyoma, uterine artery embolization (uae)

## Abstract

A woman in her 20s with no past medical history presented to the emergency department with a 4-day history of abdominal pain. Imaging revealed several large uterine fibroids that compressed various intra-abdominal structures. Options of observation, medical management, surgical management with abdominal myomectomy, and uterine artery embolization (UAE) were discussed. The patient was counseled about the associated risks of UAE and myomectomy. Since both procedures have a risk of infertility, the patient elected to proceed with uterine artery embolization due to the less invasive nature of the procedure. She was discharged after one day in the hospital following the procedure and readmitted three days later for suspected endometritis. The patient was treated with antibiotics for five days and discharged home. Eleven months post-procedure, the patient became pregnant. The patient had achieved a full-term delivery at 39 weeks and two days via a cesarean section secondary to a breech presentation.

## Introduction

Fibroids are benign, hormone-sensitive neoplasms of uterine smooth muscle that affect women of reproductive age [1]. They can have a considerable impact on women's well-being and quality of life [1]. Several risk factors have been established, most notably nulliparity and the age of menarche. Although they are one of the most common diseases in women worldwide, disparities in access to established primary care may cause delays in diagnosis and lead to worse outcomes. Symptomatology is determined by the proportion, location, and number of uterine leiomyomas. A large proportion of fibroids are asymptomatic but may present as features of mass effects such as bowel or urinary dysfunction and abdominal, pelvic, or lower back pain [2]. Symptomatic fibroids may result in abnormal uterine bleeding, infertility, and obstetric and reproductive abnormalities. Fibroids may vary greatly in size, with giant uterine fibroids presenting in a small proportion of women [3]. Depending on the patient's presentation, nature of the disease, and the desire to preserve fertility, several options are available for fibroids management [2].

## Case presentation

A 29-year-old Hispanic female with no past medical history presented to the emergency department (ED) with a four-day history of abdominal pain. She reports having a hard mass in the right abdominal region for years. The patient reported that she had never been pregnant (Gravida 0 Para 0). The patient was not sexually active nor had a partner. In the past year, she has had increasing discomfort and abdominal girth, particularly on the right side. She reports that the mass has increased significantly over the past four months. She also reported a small amount of clear vaginal discharge. On physical examination, the patient was awake, alert, and in no distress. Her vital signs revealed a temperature of 36.9 C, a heart rate of 80 beats per minute, and a blood pressure of 119/74 mm/Hg. The cardiopulmonary examination was unremarkable. Abdominal examination revealed a large, firm mass on the right side of the abdomen extending from the pubis area to the right upper quadrant.

Investigations

**Figure 1:**
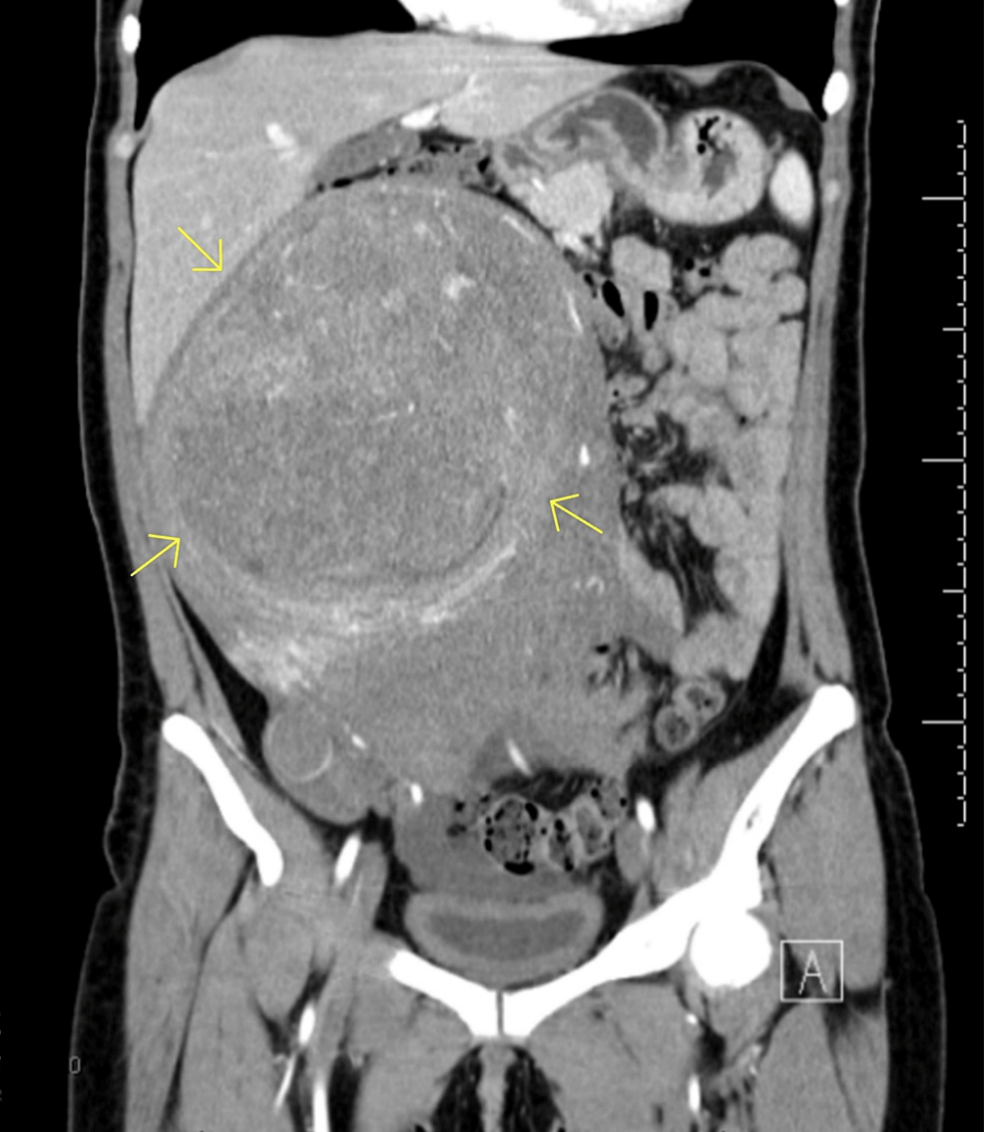
Computed tomography scan displaying a coronal view of the abdomen and pelvis exhibiting an intra-abdominal/pelvic mass (depicted by the yellow arrows) appearing to originate from the uterine fundus

**Figure 2:**
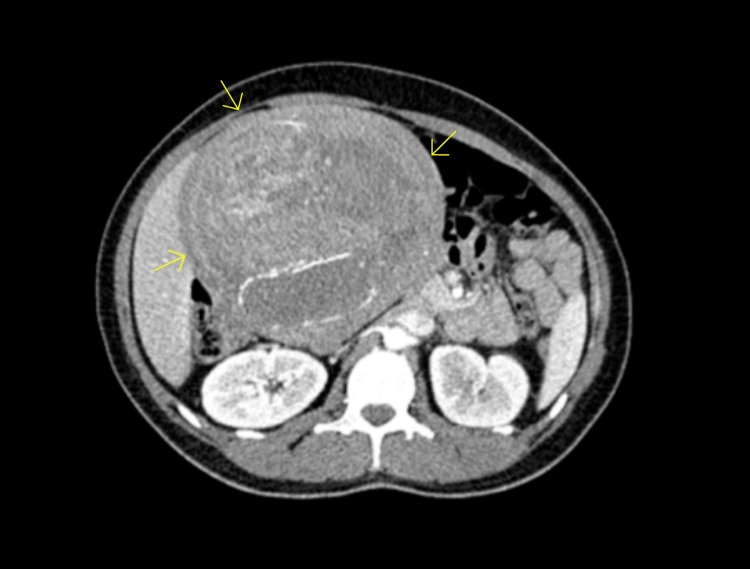
Computed tomography scan displaying an axial view of the abdomen and pelvis exhibiting an intra-abdominal/pelvic mass (depicted by the yellow arrows) appearing to originate from the uterine fundus

**Figure 3:**
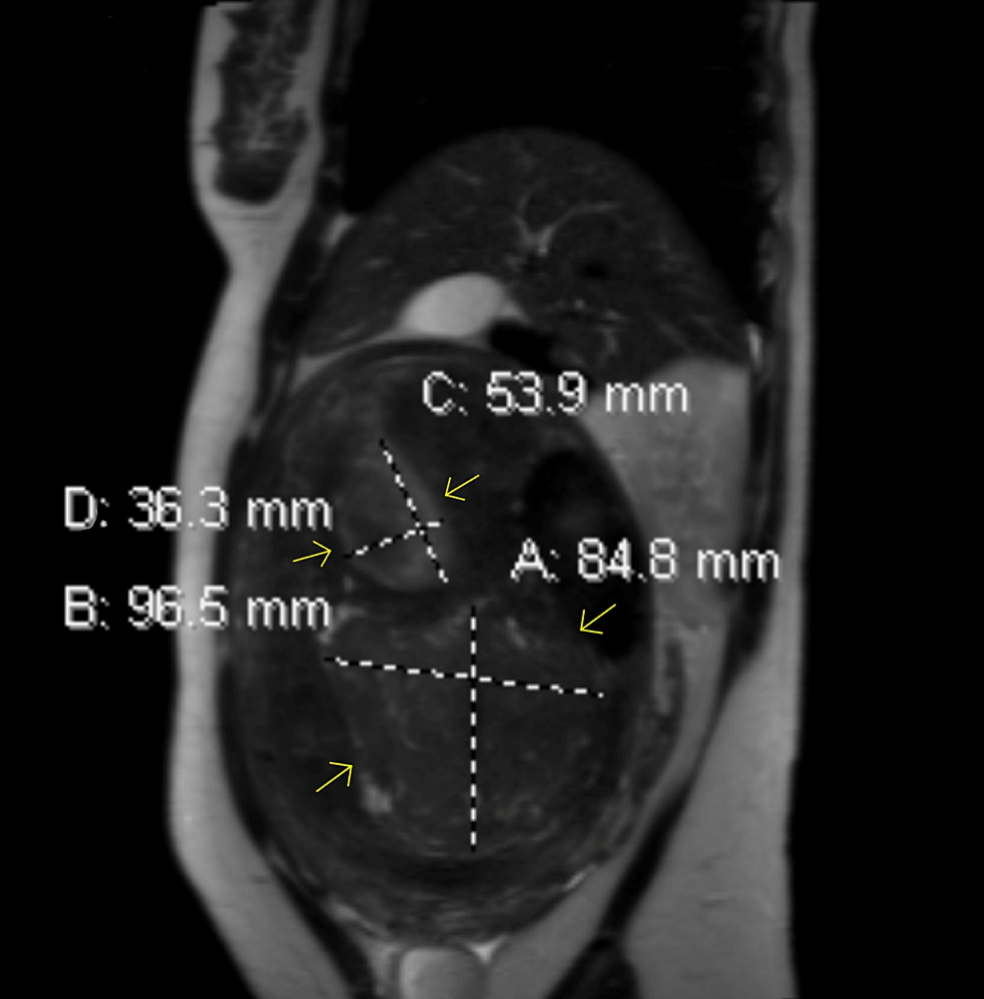
Magnetic resonance imaging displaying a sagittal view of the abdomen revealing multiple intramural fibroids (depicted by the yellow arrows) and their measurements in millimeters

The patient's laboratory studies were remarkable for a hemoglobin of 11.0 g/dL and a hematocrit of 35.6%. The chemistry panel was unremarkable. Her alpha-fetoprotein (AFP) was 4.4 ng/mL, cancer antigen 125 (CA-125) was 18 unit/mL, and carcinoembryonic antigen (CEA) was 0.4 ng/mL. A computed tomography scan of the abdomen and pelvis obtained in the emergency department detected an intra-abdominal/pelvic mass (18.0 x 14.5 x 25.2 cm) that appeared to originate from the uterine fundus. The mass was against the rectus muscles and compressed the colon, duodenum, loops of jejunum, aorta, pancreas, right kidney, and liver (Figures 1, 2). Areas of hypo-enhancement on imaging were possibly centers of necrosis. Transvaginal ultrasound revealed a large heterogeneous pelvic mass (16.4 x 13 x 16.7 cm) arising from the posterior uterine wall, raising suspicion for an intramural uterine fibroid. Magnetic resonance imaging of the abdomen with and without intravenous (IV) contrast revealed multiple intramural fibroids, some of which demonstrated hemorrhagic or myxoid/cystic changes that likely represented fibroid degeneration (Figure 3). A pap smear was obtained, returning negative for intraepithelial lesions, malignancy, and human papillomavirus (HPV). An endometrial biopsy had also been ordered, returning negative for malignancy. Additional relevant laboratory studies are shown in Table 1.

**Table 1:** Relevant laboratory results upon investigation AFP - alpha-fetoprotein, CA-125 - cancer antigen 125, CEA - carcinoembryonic antigen

Test	Patients' value	Normal value
Hemoglobin	11.0 g/dL	12.0-16.0
Hematocrit	35.6%	36%-46%
AFP	4.4 ng/mL	0-8.5
CA 125	18 unit/mL	0-35
CEA	0.4 ng/mL	0-2.5

Treatment

The large uterine fibroid caused intestinal compression in addition to significant bulk symptoms and pain. Considering the symptoms, size, and mass effect on surrounding organs, the patient was counseled on the management of the fibroid uterus. Options of observation, medical management, fertility-preserving surgical management with abdominal myomectomy, and UAE were discussed. Surgical management with abdominal myomectomy was recommended, as it was considered the most likely procedure to resolve the compressive sequelae of the fibroids and successfully mitigate the patient's symptoms. The patient was counseled on the associated risks of both UAE and myomectomy. Since both procedures have a risk of infertility, the patient elected to proceed with uterine fibroid embolization due to the procedure being less invasive. This procedure involves the injection of small sand-like particles that serve as embolic agents into arteries that supply the fibroid [4]. These particles adhere to the vessel wall, forming a clot and restricting distal blood supply, subsequently resulting in the shrinking of the fibroid.

Outcome and follow-up

**Figure 4:**
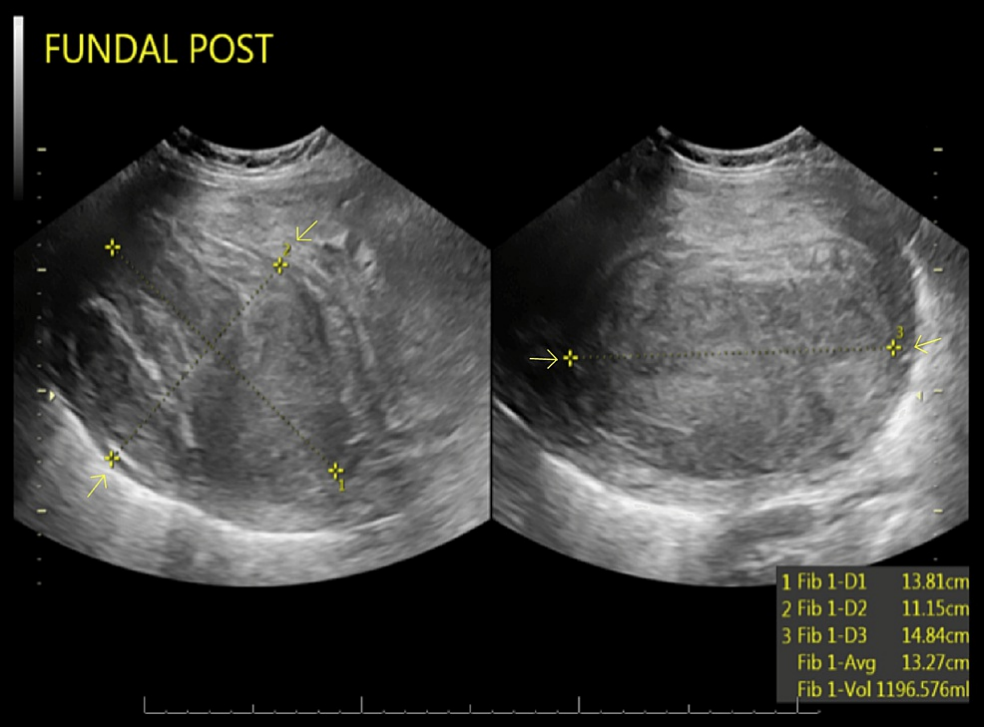
Second-trimester transvaginal ultrasound exhibiting sagittal and transverse views of the patients' largest fibroid (depicted by the yellow arrows)

The patient successfully underwent UAE without complications and tolerated it well. She remained in the hospital for one day and was discharged with instructions to follow up with interventional radiology after two weeks and gynecology after four weeks. She was also counseled on contraception use for three months post-procedure and was given a prescription for oral contraceptives. After two days, she presented again to the ED with complaints of worsening right-sided abdominal pain, fever of 39.2 C, tachycardia, and vaginal spotting. Physical examination revealed abdominal tenderness to palpation, and laboratory studies exhibited a white blood cell count of 12.6 x 10^3^/mcl (normal range 4.0-11.0 x10^3^/mcl) with left shift and C-reactive protein elevated to 35.7 mg/dL (normal value <0.3 mg/dL). MRI of the abdomen and pelvis with IV contrast demonstrated findings that showed multiple uterine fibroids with curvilinear lucent areas within the uterus, representing post-embolization changes. Given her current presentation, the patient was readmitted for suspected endometritis and started on empiric intravenous antibiotics with ampicillin, gentamicin, and clindamycin. Endometritis is a rare complication of UAE [5]. Transvaginal ultrasound was repeated and revealed decreased fibroid size post UAE compared to the prior ultrasound. During hospitalization, she occasionally had a brown vaginal discharge. After five days of hospitalization, the patient's fever and leukocytosis resolved. The antibiotics were changed to oral doxycycline and metronidazole, taken twice daily for 14 days. She was discharged on the sixth day with a course of antibiotics and a follow-up appointment. The patient was seen four weeks following the procedure in the gynecology clinic. She had completed the antibiotics and had reported significant improvement in her inflammation and pain symptoms. Eleven months post-procedure, the patient had become pregnant. Six months into her pregnancy, transvaginal ultrasound showed the largest posterior fundal fibroid with a measurement of 13.81 x 11.15 x 14.84 cm (Figure 4). The patient had achieved a full-term delivery at 39 weeks and two days via a cesarean section secondary to breech presentation.

## Discussion

Fibroids affect up to 80% of women by the age of 50, being the leading cause of hysterectomy in the United States and inflicting a financial burden of more than 30 billion dollars annually [6]. It is for these reasons that the most effective and affordable methods of treating fibroids should be outlined. Surgical management with a hysterectomy is effective for treatment but may not be the best option for women desiring fertility preservation. UAE was ﬁrst used to treat uterine ﬁbroids in 1995 as an alternative to a hysterectomy [7]. Embolization of the uterine artery results in ischemic necrosis of uterine fibroids, while the myometrium becomes revascularized [4,7].

Options of treatment in large symptomatic fibroids include observation, medical management, abdominal myomectomy, and UAE. Observation involves no intervention and would likely lead to further fibroid growth over time. Medical management with gonadotropin-releasing hormone agonists (GnRH) has also been shown to reduce fibroid size, but given the size of our patient's fibroids, it would likely have been ineffective. Fertility-preserving surgery with an abdominal myomectomy involves a midline vertical incision and focuses on the largest fibroids causing symptoms. However, it carries the possible risk of a hysterectomy as a lifesaving measure in the setting of a hemorrhage. Other risks of a myomectomy include bleeding, infection, and damage to nearby organs. UAE carries similar risks, including infection, bleeding, intra-abdominal hemorrhage, post-procedure obstructing fibroid with endometritis, need for a hysterectomy, and infertility.

One of the feared complications in the treatment of large fibroids is loss of fertility [7]. Our patient had never been pregnant (Gravida 0), did not have a partner, nor was she sexually active or desired to become pregnant in the near future. However, the patient expressed that she wanted to preserve her fertility. The patient was counseled on the associated risks of UAE and myomectomy. She was informed that both procedures have a risk of infertility. After understanding the risks and benefits, the patient elected to proceed with uterine fibroid embolization due to the less invasive nature of the procedure. According to the American College of Obstetricians and Gynecologists Committee on Practice Bulletins, UAE is a procedure in treating uterine leiomyomas that is recommended for patients who seek uterine preservation, provided that they are advised about the minimal data available in regard to reproductive outcomes [8]. They acknowledge that there remains conflicting evidence on the reproductive outcomes of UAE in comparison to myomectomy, as the current studies in the literature contain small sample sizes in which comparative conclusions are difficult to deduce [8]. Once an appropriate discussion of the benefits, risks, and alternatives to UAE has been communicated with the patient, any woman who prefers to avoid undergoing surgery in the treatment of this condition should still be offered the option of undergoing UAE, even if future fertility is desired [9]. 

Thus, in practice, UAE is often not the first procedure recommended for women who wish to preserve fertility, as the effect of UAE on future pregnancy still remains uncertain. While this option may reduce the size of her fibroids, it may also place her at risk for infertility. However, recent publications suggest that obstetrical success was indeed possible in cases involving complete necrosis of treated lesions in which uterine anatomy restoration was achieved [10]. This same study demonstrated that among 398 female patients who had undergone a prior UAE procedure, 148 pregnancies and 109 live births had been documented [10].

In a randomized controlled trial, Donnez and coworkers showed that the reduction of symptoms and quality of life post-UAE is similar to that of hysterectomy, with the advantage of a shorter hospitalization and faster recovery. Reoperation, a disadvantage of UAE, occurs in 15-20% of cases in which embolization was successful and in 50% of cases with incomplete infarction [7]. The authors showed that myomectomy was more favorable than UAE for pregnancy rate (78% vs. 50%), delivery rate (48% vs. 19%), and abortion rate (23% vs. 64%) [7].

In our patient, an MRI of the pelvis and gynecological US was performed one week after embolization, displaying decreasing sizes of the fibroids. Follow-up MRI imaging revealed approximately a 20% reduction in overall uterine size with a decrease in fibroid size, as expected, following UAE. The pre-procedure uterus dimensions were 23 x 14.7 x 17.7 cm, and the post-procedure uterine dimensions were 20 x 15 x 16 cm, respectively. It is important to note that a larger fibroid size does not negatively affect the efficacy of this procedure, as there are several reports which demonstrate the pronounced safety and efficacy of UAE in patients with fibroids that are larger than 10 cm [9]. However, intra-abdominal hemorrhage can occur in some cases in patients with large fibroids [11]. Fortunately, in our patient, imaging was negative for intra-abdominal hemorrhage. 

The possibility of malignancy was strongly considered during the management of our patient. The patient had no prior medical or family history of gynecological malignancy. A pap smear carried out prior to the UAE procedure returned negative for intraepithelial lesions, malignancy, and HPV. In addition, endometrial biopsy had returned negative for malignant cells. The mass was likely a fibroid, given the negative tumor markers, imaging findings, family history, and lack of symptoms that are concerning for malignancy.

## Conclusions

Of the several therapeutic options for uterine fibroids, including both medical and surgical management, the treatment of uterine fibroids should be individualized according to a patient's fibroid size and desire for fertility preservation. While UAE is one of the less invasive options for treating fibroids, it is important that patients are educated on the potential risks to fertility following UAE. In addition to the risk of infertility after this procedure, patients should be aware of the possible risks that may arise immediately afterward. These risks include post-procedure infection and significant pain following UAE. While infertility risks are still of concern, patients should not be denied the option of this minimally invasive procedure. Patients should be encouraged to follow up accordingly if concerning symptoms arise for further workup and treatment. With proper follow-up and post-procedure care, UAE is a minimally invasive treatment option for patients with large fibroids to reduce fibroid size, pain, and distressing symptoms.
